# Supplementary material for: Dissociating Autoantibody Responses against Ro52 Antigen in Patients with Anti-Synthetase or Anti-MDA5 Antibodies
Source: Diagnostics (Basel). 2023 Dec 8;13(24):3621. doi: 10.3390/diagnostics13243621 (PMC10742448; doi:10.3390/diagnostics13243621)
Supplement: Supplementary file 1 [file diagnostics-13-03621-s001.zip › diagnostics-2732684-supplementary.pdf]

(A)

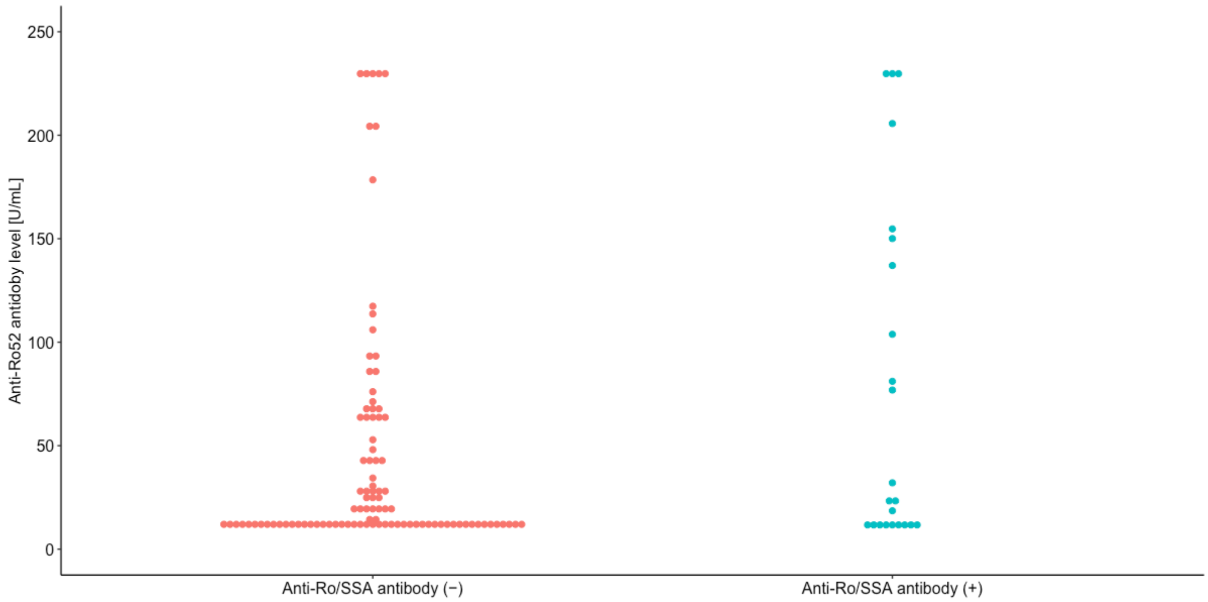

(B)

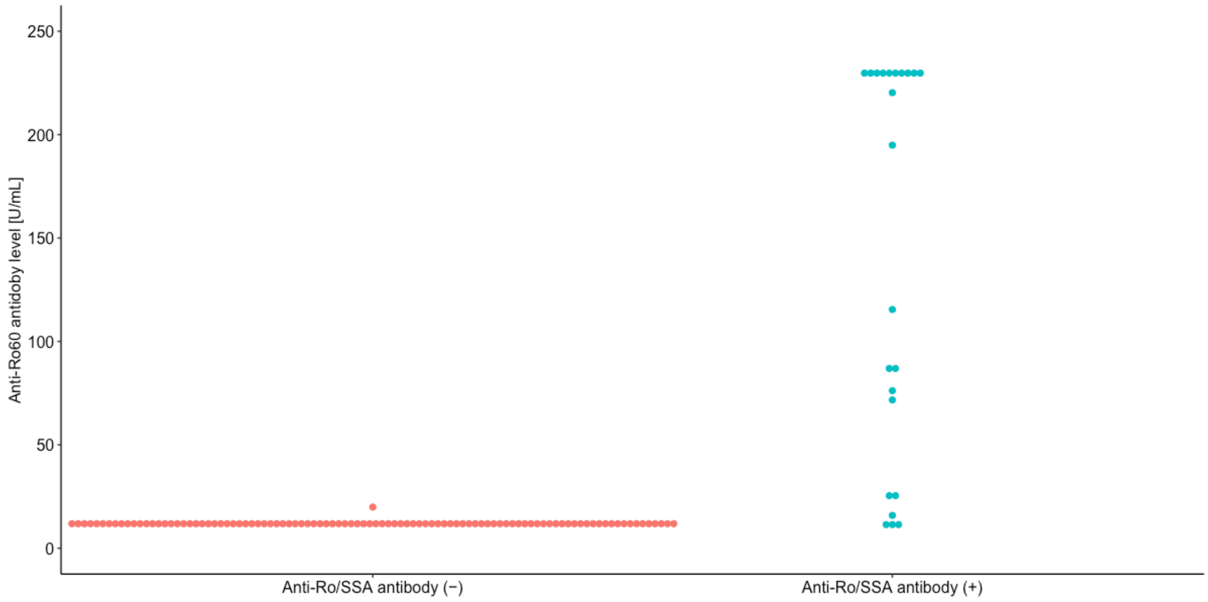

Supplementary Figure S1. (A) Anti-Ro52 antibody levels and **(B)** Anti-Ro60 antibody levels stratified by anti-Ro/SSA antibody-positivity by RNA-IP in patients with anti-synthetase antibodies.

(A)

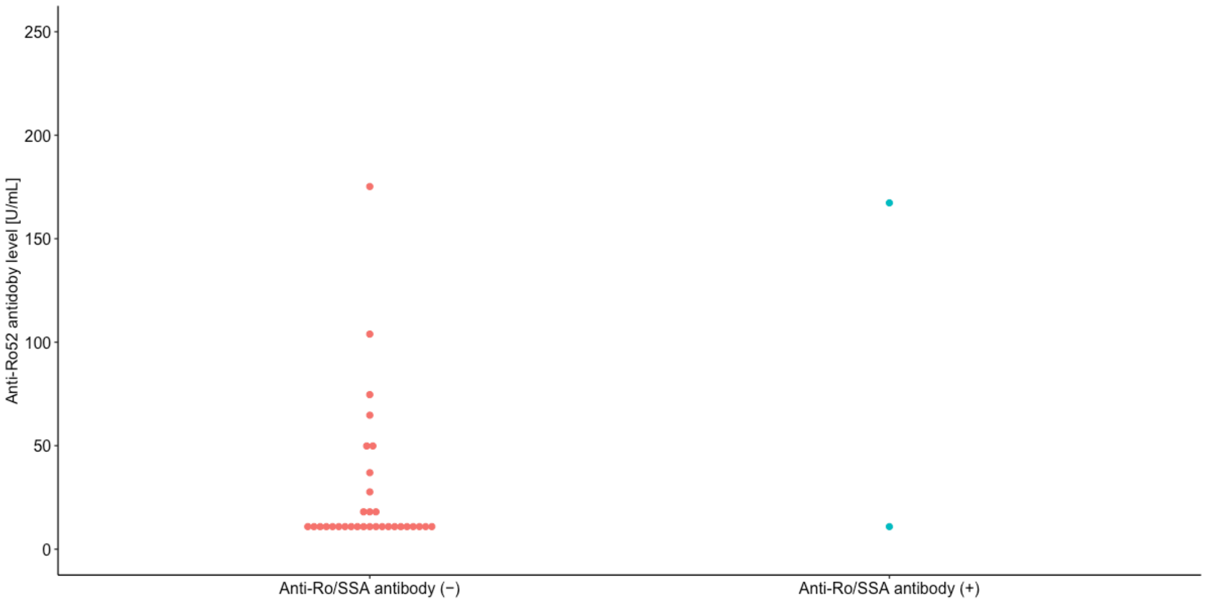

(B)

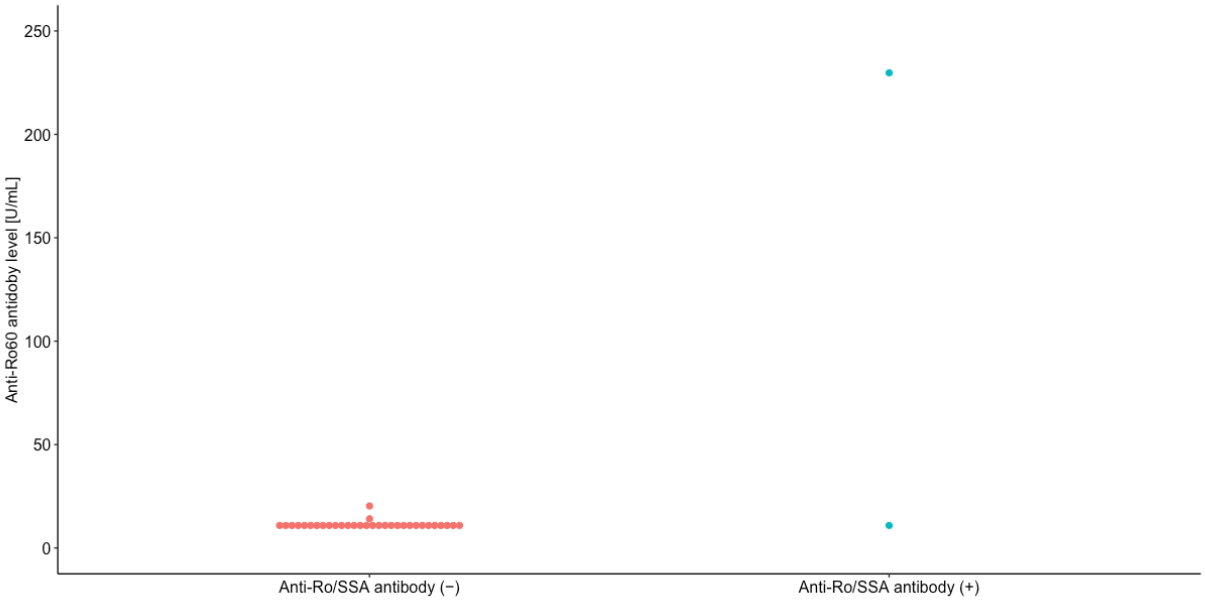

Supplementary Figure S2. (A) Anti-Ro52 antibody levels and **(B)** Anti-Ro60 antibody levels stratified by anti-Ro/SSA antibody-positivity by RNA-IP in patients with anti-MDA5 antibodies.

Supplementary Table S1. Previous studies reporting the prevalence and clinical significance of anti-Ro52 antibodies in patients with anti-synthetase antibodies

Study	Country	Study type	Sample size	Anti-Ro52 antibody testing method	Prevalence of anti-Ro52 antibodies	Clinical association
Brouwer *et al*. [36]	11 European countries	Retrospective	85	EIA	65.9%	Not reported
Dugar *et al*. [37]	Australia	Retrospective	42	EIA	35.7%	No association
Marie *et al*. [14]	France	Retrospective	89	EIA	40.4%	Symptomatic ILD<br>Malignancy, poor OS
Yamasaki *et al*. [38]	Japan	Retrospective	97	EIA	59.0%	Overlap syndrome
Bauhammer *et al*. [15]	Germany	Retrospective	61	LIA	42.9%	Acute ILD<br>Non-response to IS drugs
Shi *et al*. [16]	China	Retrospective	124	LIA	29.0%	RP-ILD
Pinal-Fernandez *et al*. [17]	USA	Retrospective	169	LIA	65.7%	Arthritis, mechanic's hands, DM-specific rash<br>No association with malignancy or poor OS
Sreevilasan *et al*. [39]	India	Retrospective	28	LIA	50.0%	No association with ILD
Bozzalla-Cassione *et al*. [18]	Italy	Retrospective	60	LIA	56.7%	ILD, myositis<br>No association with poor OS

Gui *et al*. [13]	China	Retrospective	173	LIA	53.2%	No association with RP-ILD or poor OS
Martins *et al*. [40]	Portugal	Retrospective	70	LIA	21.4%	No association
Sodsri *et al*. [41]	Thailand	Retrospective	82	LIA	45.1%	ILD, poor OS

DM, dermatomyositis; EIA, enzyme immunoassay; ILD, interstitial lung disease; IS, immunosuppressive; LIA, line immunoassay; NA, not available; OS, overall survival; RP-ILD, rapidly progressive interstitial lung disease.

Supplementary Table S2. Previous studies reporting the prevalence and clinical significance of anti-Ro52 antibodies in patients with anti-MDA5 antibodies

Study	Country	Study type	Sample size	Anti-Ro52 antibody testing method	Prevalence of anti-Ro52 antibodies	Clinical association
Huang *et al*. [19]	Canada	Retrospective	21	LIA	61.9%	RP-ILD<br>Skin ulceration
Xu *et al*. [20]	China	Retrospective	83	LIA	74.7%	RP-ILD<br>Skin ulceration<br>Poor OS
Cavagna *et al*. [21]	Italy	Retrospective	149	LIA	31.5%	RP-ILD
Gui *et al*. [13]	China	Retrospective	173	LIA	53.2%	RP-ILD<br>Poor OS
You *et al*. [22]	China	Retrospective	272	LIA	64.0%	RP-ILD
Wang *et al*. [23]	China	Retrospective	126	LIA	60.3%	Poor OS
Liu *et al*. [24]	China	Retrospective	200	LIA	63.3%	Poor OS

LIA, line immunoassay; OS, overall survival; RP-ILD, rapidly progressive interstitial lung disease.
